# Comparative proteomic analysis of somatic embryo maturation in *Carica papaya* L.

**DOI:** 10.1186/1477-5956-12-37

**Published:** 2014-06-26

**Authors:** Ellen de Moura Vale, Angelo Schuabb Heringer, Tatiana Barroso, André Teixeira da Silva Ferreira, Monique Nunes da Costa, Jonas Enrique Aguilar Perales, Claudete Santa-Catarina, Vanildo Silveira

**Affiliations:** 1 Laboratório de Biotecnologia, Centro de Biociências e Biotecnologia (CBB), Universidade Estadual do Norte Fluminense Darcy Ribeiro (UENF), Av. Alberto Lamego 2000, Campos dos Goytacazes, RJ 28013-602, Brazil; 2 Laboratório de Toxinologia da Fundação Oswaldo Cruz, Instituto Oswaldo Cruz. Instituto Oswaldo Cruz /IOC /FIOCRUZ, Av. Brasil, 4365. Manguinhos, CEP: 21040-360 Rio de Janeiro, RJ, Brazil; 3 Laboratório de Biologia Celular e Tecidual, CBB-UENF, Av. Alberto Lamego 2000, Campos dos Goytacazes, RJ 28013-602, Brazil

**Keywords:** Papaya, Somatic embryogenesis, Protein profile, Mass spectrometry, Two-dimensional electrophoresis

## Abstract

**Background:**

Somatic embryogenesis is a complex process regulated by numerous factors. The identification of proteins that are differentially expressed during plant development could result in the development of molecular markers of plant metabolism and provide information contributing to the monitoring and understanding of different biological responses. In addition, the identification of molecular markers could lead to the optimization of protocols allowing the use of biotechnology for papaya propagation and reproduction. This work aimed to investigate the effects of polyethylene glycol (PEG) on somatic embryo development and the protein expression profile during somatic embryo maturation in papaya (*Carica papaya* L.).

**Results:**

The maturation treatment supplemented with 6% PEG (PEG6) resulted in the greatest number of somatic embryos and induced differential protein expression compared with cultures grown under the control treatment. Among 135 spots selected for MS/MS analysis, 76 spots were successfully identified, 38 of which were common to both treatments, while 14 spots were unique to the control treatment, and 24 spots were unique to the PEG6 treatment. The identified proteins were assigned to seven categories or were unclassified. The most representative class of proteins observed in the control treatment was associated with the stress response (25.8%), while those under PEG6 treatment were carbohydrate and energy metabolism (18.4%) and the stress response (18.4%).

**Conclusions:**

The differential expression of three proteins (enolase, esterase and ADH3) induced by PEG6 treatment could play an important role in maturation, and these proteins could be characterized as candidate biomarkers of somatic embryogenesis in papaya.

## Background

Papaya (*Carica papaya* L. - Caricaceae) is one of the most commonly cultivated fruit trees in tropical and subtropical regions and one of the most frequently consumed fruits in the world [1]. Since the papaya genome was sequenced, some of the identified features have made papaya an excellent model system for studying tropical plants [2,3], including proteomic studies [4]. A good model system in biology should be easy to grow and maintain, have a strong genetic and genomic foundation and be in a suitable position phylogenetically for comparative studies [2].

A recurrent problem in the cultivation of this species has been seed propagation, which generates heterogeneous plants and results in a reduction of fruit production [5]. One solution for increasing the production of this species involves the cloning of elite cultivars that generate more uniform plants. Somatic embryogenesis, a biotechnological tool, has the potential to achieve high multiplication rates to scale up production.

Somatic embryogenesis was first induced in papaya by Bruijne et al. [6], and many efforts have since been made to optimize the process. These protocols have been hampered due to the numerous variables that must be regulated during the process, including the endogenous levels of phytohormones, proteins and genetic and epigenetic factors [7]. Maturation is a critical process during embryo development for the conversion of somatic embryos into plantlets [8]. This stage is characterized by cellular expansion, differentiation and the accumulation of reserve substances and is therefore a key determinant of successful somatic embryo regeneration and the conversion of somatic embryos into plantlets [9].

Water stress, such as that produced by polyethylene glycol (PEG), has been proposed to be an important factor during the maturation of somatic embryos [10-13] and is potentially able to unleash rapid biochemical changes in the activity of specific proteins during maturation [14]. These high-molecular-weight molecules are not able to pass through the cell wall, which leads to a restriction of water absorption, low turgor pressure and a reduction in the intracellular osmotic potential [10], causing desiccation. PEG has been used as a maturation agent in various species, including *Hevea brasiliensis*[15], *Glycine max*[16], *Aesculus hippocastanum*[17], *Prosopis laevigata*[18] and *C. papaya*[19,20].

Proteomic studies in plants have resulted in advances in knowledge with regard to the structure, genetic organization and evolution of plant genomes [21]. Proteomic studies in papaya are still scarce but have been increasing in recent years [4,22-25]. However, no reports currently exist that relate protein levels to the development and control of somatic embryogenesis in papaya.

Many studies have been conducted using this tool to understand the mechanisms that control somatic embryogenesis in various species, such as *Vitis vinifera*[26], *Phoenix dactylifera*[27], *Cyclamen persicum*[28], *Theobroma cacao*[29], *Crocus sativus*[30] and *Quercus suber*[31]. The determination of dynamic changes in protein concentrations during embryonic development could lead to improvements in somatic embryogenesis protocols that would allow the use of biotechnological tools for papaya propagation and breeding. The present study investigated the effects of PEG on somatic embryo development and on the protein expression profile during somatic embryo maturation in papaya.

## Results and discussion

### Maturation and conversion of somatic embryos

**Figure 1:**
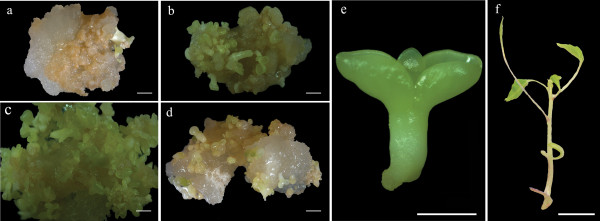
**Morphological characteristics of embryogenic cultures of *C. papaya *subjected to maturation treatments (control) (A); (PEG3) (B); (PEG6) (C) and (PEG9) (D).** Morphological characteristics of cotyledonary (mature) somatic embryos **(E)**; and regenerated plantlets **(F)**. PEG: polyethylene glycol; Bars: **(A-D)**: 0.5 mm; **(E)**: 0.2 mm and **(F)**: 15 mm.

A significant effect of PEG was observed with regard to the maturation of somatic papaya embryos after 42 days of culture (Table 1 and Figure 1). The cultures incubated under the control and PEG6 treatments yielded 15 and 40 cotyledonary somatic embryos per colony (Table 1), respectively. The PEG6 treatment enabled the conversion of the somatic embryos into plantlets with leaves and stems, similar to those with a seed origin (Figure 1).

**Table 1:** Number of cotyledonary somatic embryos (per 300 mg of cells initially inoculated), percentage of dry matter (DM) and the protein content in embryogenic cultures of the *C. papaya* hybrid UENF/CALIMAN01 after 42 days of incubation under the different maturation treatments

**Variables**	**PEG (%)**			
	**0**	**3**	**6**	**9**
**NSE**	15.0 b*	20.0 b	40.0 a	23.0 b
**DM (%)**	11.0 b	12.9 b	16.2 a	17.1 a
**Protein (mg/g DM)**	50.7 a	49.8 a	62.2 a	2.0 b

*Means followed by the same letters are not significantly different according to the SNK test (P < 0.05). NSE: C.V.(%) = 26.7, n = 4; DM: C.V.(%) = 9.9, n = 4; Protein (mg/g DM): C.V.(%) = 18.3, n = 3. PEG - polyethylene glycol; DM - dry matter; NSE - number of somatic embryos.

There were significant differences in the percentage of dry matter (DM) in the cultures subjected to different concentrations of PEG. The cultures matured under 6 and 9% PEG showed the highest percentages of DM (Table 1). These results indicate that PEG promoted an increase in the DM content of the cultures.

According to the obtained results, the addition of PEG significantly affected the maturation of somatic embryos as well as the protein concentrations and profiles in these embryos during somatic embryogenesis in the *C. papaya* hybrid UENF/CALIMAN01. PEG6 treatment resulted in the greatest numbers of cotyledonary somatic embryos (Table 1). Similar results were previously described during somatic embryo maturation in this papaya hybrid [20], where the authors observed increased promotion of the maturation of embryogenic cultures when using PEG compared with the control treatment (i.e., without PEG). Koehler et al. [32] found that supplementation with 5% PEG combined with 2 g/L activated charcoal and 5 μM abscisic acid (ABA) improved the quality of somatic embryos and increased the formation of plantlets. These authors concluded that PEG was an important inducer of maturation that aided in the conversion of somatic papaya embryos.

Maturation is a crucial phase during somatic embryogenesis, and the addition of maturation promoters such as PEG, ABA and activated charcoal is crucial for promoting the maturation of somatic embryos and for their conversion into plantlets [33].

PEG is a non-plasmolysing osmoticum that is unable to readily penetrate plant cell walls and produces an *in vitro* effect similar to that of natural water stress during zygotic embryogenesis [12]. PEG has been shown to enhance the accumulation of storage reserves and increase desiccation tolerance in cotyledonary somatic embryos, and it has also been shown to have a slight impact on endogenous ABA levels in angiosperms and conifers [10-12]. PEG has been reported to act by controlling the expression of several genes involved in embryonic differentiation and the development of apical meristems [13].

During the development of the zygotic embryo, desiccation and the accumulation of reserve substances have been directly related to the differential expression of genes and metabolic pathways, which are critical processes for the successful development and germination of the embryo [9]. The main storage reserves found in the seeds of papaya are lipids (28.8) and proteins (27.8), while the seeds are poor in free monosaccharides, with sucrose being the main sugar present [34]. Somatic embryogenesis, which mimics zygotic embryo development, has been considered as a model for studying zygotic embryogenesis. Studies have been conducted to shed light on embryonic development in many species, such as *Citrus sinensis*[35], *Picea* sp. [36,37] and *Cyclamen* sp. [38].

The maturation of somatic embryos also appears to be highly dependent on stress, such as that induced by PEG. Stress modifies DNA methylation patterns and induces the synthesis of proteins that are essential to embryonic development, enabling embryos to mature [39], as observed in the present work.

**Figure 2:**
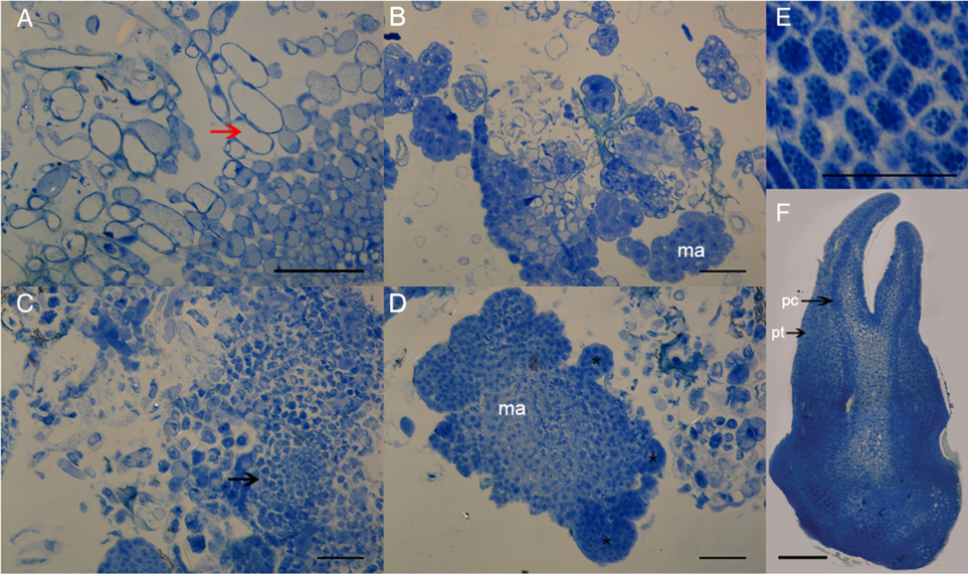
**Protein staining with Coomassie brilliant blue R-250 (CBB) in embryogenic cultures of *C. papaya *after 42 days of incubation in the control (without PEG) and PEG6 (with 6% PEG) maturation treatments. (A-B)** Control treatment; **(C-D)** PEG6 treatment; **(E)** protein granules in embryogenic cells; **(F)** cotyledonary somatic embryo under PEG6 treatment. *Red arrow*: non-embryogenic cells; *black arrows*: embryogenic cells, *ma*: meristematic aggregates; *Asterisks* (*): proembryo; *pt*: protoderm; *pr*: procambium. PEG: polyethylene glycol. Bars: 200 μm.

Histomorphological sections revealed that cultures subjected to PEG6 treatment presented more cells with embryogenic characteristics. PEG induces a high proportion of small isodiametric cells with dense cytoplasm and large nuclei, organized in meristematic aggregates (Figure 2), as previously described in embryogenic cultures of papaya [40]. Histochemical analysis of these cultures showed that cells with embryogenic characteristics, meristematic aggregates and somatic embryos exhibited an intense reaction to the protein dye CBB (Figure 2). Proteins were visualized in the form of granules in embryos from early stages, as in cotyledonary embryos (Figure 2E, F). In cotyledonary embryos, the proteins were more abundant in the basal region of the embryo (Figure 2F).

In this context, the increase in protein content observed in cultures incubated under PEG6 treatment is associated with large amounts of protein bodies identified in meristematic cells and somatic embryo tissues, which were more abundant in this treatment. The PEG-induced proteins could be an important factor in somatic embryo development and posterior plantlet conversion. The synthesis of specific proteins, such as storage proteins, is known to be crucial for *in vitro* morphogenesis, serving as the principal nitrogen source during seed germination [41,42].

### Proteomic analysis

**Figure 3:**
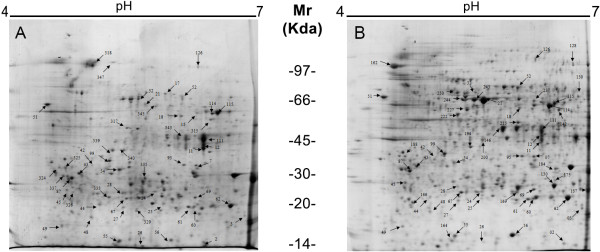
**Two-dimensional gel electrophoresis (2-DE) reference maps of embryogenic cultures of the *C. papaya *after 42 days of incubation in the (A) control (without PEG) and (B) PEG6 (with 6% PEG) maturation treatments.** PEG: polyethylene glycol

Significant differences in protein concentrations were observed between the different treatments (Table 1). The PEG9 treatment induced a reduce in soluble proteins (2.0 mg of proteins/g of DM), and there were no statistically significant differences between control, PEG3 and PEG 6 treatments (Table 1). These results indicate that under the condition of 9% PEG, the stress was so high that cell metabolism was reduced and protein synthesis was affected.Because PEG6 was determined to be the best treatment for the development of mature somatic embryos and led to the highest protein content, the 2-DE profiles of embryogenic cultures from the control and PEG6 treatments were analyzed (Figure 3). The numbers of spots obtained for the control and PEG6 treatments were 422 and 688, respectively, while 217 spots were common to the two treatments.

In addition to being the best treatment for achieving somatic embryo maturation (Table 1), PEG6 induced a change in the 2-DE profile (Figure 3) and number of identified spots compared with the control treatment. The actions of PEG as a maturation agent could be related to the induction of specific proteins associated with the significant dehydration process observed during late embryogenesis, or the PEG-induced changes could be related to the synthesis of storage proteins promoted by water stress [10]. In this context, analyses of molecular and biochemical changes, such as the protein profiles induced by PEG, can provide important information for understanding somatic embryogenesis because proteins play an essential role during somatic embryo histodifferentiation [43]. Additionally, proteins represent the main reserves that accumulate during embryonic development in most species [44].

Of the 135 protein spots selected for MS/MS analysis, 76 were successfully identified, and 38 proteins were common to the two treatments. Among these proteins, 14 were up-regulated, while 3 were down-regulated in the control versus the PEG6 treatment. Additionally, 14 spots were unique to the control treatment, and 24 spots were unique to the PEG6 treatment. Representative graphs of the mean percentage volumes (%vol) of each of these spots are provided in Table 2. In the present study, identification of the proteins was relied essentially on homology search to known sequences of the other plant species because of the poor protein sequence information that is currently available for *Carica Papaya* at the NCBInr database.

**Table 2:** **Expressed proteins identified via mass spectrometry in the embryogenic cultures of the *C. papaya *** **after 42 days in maturation treatments**

**Spot**^**a**^	**Tratament**^**b**^	**Function**^**c**^	**Accession number**^**d**^	**Protein name**	**Organism**	**Peptides number**^**e**^	**Coverage (%)**^**f**^	**Score**^**g**^	**Abundance**^**h**^
									**Control PEG6**
**Proteins of higher abundance in PEG6 treatment**									
017	Control/PEG6	Stress response, glycolysis	gi|3914394	2,3-bisphosphoglycerate-independent phosphoglycerate mutase	*Mesembryanthemum crystallinum*	4	8%	196	
021	Control/PEG6	Carbohydrate and energy metabolism	gi|3023685	Full = Enolase	*Alnus glutinosa*	3	10%	199	
114	Control/PEG6	Stress response	gi|211906436	UDP-D-glucose pyrophosphorylase	*Gossypium hirsutum*	4	14%	363	
128	PEG6	Stress response, Others functions	gi|151347486	Methionine synthase	*Carica papaya*	3	5%	70	
130	PEG6	Metabolic process	gi|1743354	Aldehyde dehydrogenase (NAD+)	*Nicotiana tabacum*	2	4%	137	
138	PEG6	Stress response, oxidation-redution process, Carbohydrate and energy metabolism	gi|15222848	Glyceraldehyde 3-phosphate dehydrogenase	*Arabidopsis thaliana*	3	15%	147	
139	PEG6	Protein metabolism, stress response	gi|3914394	2,3-bisphosphoglycerate-independent phosphoglycerate mutase	*Mesembryanthemum crystallinum*	2	3%	68	
142	PEG6	Protein metabolism, stress response	gi|21487	Leucine aminopeptidase	*Solanum tuberosum*	1	2%	56	
146	PEG6	Oxidation-redution process	gi|29501684	Alcohol dehydrogenase 3	*Petunia x hybrida*	3	10%	136	
162	PEG6	Protein metabolism, stress response	gi|38154482	Molecular chaperone Hsp90-1	*Nicotiana benthamiana*	5	8%	189	
163	PEG6	Others functions	gi|1707372	Ubiquitin-like protein	*Arabidopsis thaliana*	3	25%	119	
165	PEG6	Unclassified	gi|20140866	Full = Translationally controlled tumor protein homolog	*Cucumis melo*	2	13%	83	
166	PEG6	Other functions	gi|295885749	Cytokinin oxidase 2	*Triticum aestivum*	1	1%	46	
169	PEG6	Fatty acid metabolism, Carbohydrate and energy metabolism	gi|136057	Full = Triosephosphate isomerase, cytosolic	*Coptis japonica*	1	3%	55	
175	PEG6	Fatty acid metabolism	gi|147784332	Hypothetical protein VITISV_041523	*Vitis vinifera*	2	9%	69	
184	PEG6	Metabolic process	gi|255565327	Esterase D, putative	*Ricinus communis*	1	6%	46	
188	PEG6	Carbohydrate and energy metabolism	gi|82621108	Phosphoglycerate kinase-like	*Solanum tuberosum*	2	8%	212	
194	PEG6	Oxidation-reduction process, fatty acid metabolism	gi|2204087	Enoyl-ACP reductase	*Arabidopsis thaliana*	2	12%	75	
200	PEG6	Seed germination, cytoskeleton organization	gi|32186896	Actin	*Gossypium hirsutum*	6	22%	454	
215	PEG6	Oxidation-redution process, stress response	gi|449458315	PREDICTED: glutamate dehydrogenase 2-like	*Cucumis sativus*	2	6%	95	
222	PEG6	Others functions	gi|38564733	Initiation factor eIF4A-15	*Helianthus annuus*	3	14%	190	
227	PEG6	Carbohydrate and energy metabolism	gi|3023685	Full = Enolase	*Alnus glutinosa*	2	8%	110	
237	PEG6	Stress response	gi|169661	S-adenosylhomocysteine hydrolase	*Petroselinum crispum*	1	8%	45	
243	PEG6	Carbohydrate and energy metabolism	gi|3023685	Full = Enolase	*Alnus glutinosa*	2	8%	130	
244	PEG6	Carbohydrate and energy metabolism	gi|3023685	Full = Enolase	*Alnus glutinosa*	2	8%	110	
250	PEG6	Unclassified	gi|462413398	Hypothetical protein PRUPE_ppa003850mg	*Prunus persica*	3	8%	130	
**Proteins of higher abundance in Control treatment**									
002	Control/PEG6	Unclassified	gi|384249809	HI0933-like protein	*Coccomyxa subellipsoidea*	1	2%	50	
011	Control/PEG6	Fatty acid metabolism	gi|257096376	Full = GDSL esterase/lipase	*Carica papaya*	2	6%	82	
012	Control/PEG6	Fatty acid metabolism	gi|257096376	Full = GDSL esterase/lipase; AltName: Full = CpEST	*Carica papaya*	2	6%	82	
018	Control/PEG6	Stress response, Carbohydrate and energy metabolism	gi|414866626	TPA: hypothetical protein ZEAMMB73_999129	*Zea mays*	2	6%	64	
025	Control/PEG6	Oxidation-redution process	gi|255555109	Flavoprotein wrbA, putative	*Ricinus communis*	1	11%	112	
026	Control/PEG6	Other functions	gi|162464222	Small GTP binding protein1	*Zea mays*	2	11%	95	
042	Control/PEG6	Carbohydrate and energy metabolism	gi|14423688	Full = Enolase 1	*Hevea brasiliensis*	2	8%	62	
043	Control/PEG6	Stress response	gi|2853219	Glutathione transferase	*Carica papaya*	5	22%	330	
048	Control/PEG6	Others functions	gi|89212810	14-3-3-like protein	*Gossypium hirsutum*	4	15%	213	
049	Control/PEG6	Protein metabolism	gi|399942	Full = Stromal 70 kDa heat shock-related protein	*Pisum sativum*	4	9%	205	
055	Control/PEG6	Other functions	gi|162464222	Small GTP binding protein1	*Zea mays*	2	11%	95	
054	Control/PEG6	Stress response, protein metabolism	gi|475610277	Aspartic proteinase oryzasin-1	*Aegilops tauschii*	1	2%	60	
111	Control/PEG6	Fatty acid metabolism	gi|257096376	Full = GDSL esterase/lipase	*Carica papaya*	6	18%	389	
115	Control/PEG6	Stress response, Carbohydrate and energy metabolism	gi|7417426	UDP-glucose pyrophosphorylase	*Oryza sativa Indica Group*	2	6%	53	
315	Control	Stress response, metabolic process	gi|32527831	UDP-glucose pyrophosphorylase	*Populus tremula x Populus tremuloides*	3	9%	122	
317	Control	Other functions	gi|225442221	Initiation factor eIF4A-15	*Vitis vinifera*	4	16%	154	
318	Control	Stress response	gi|470129411	PREDICTED: vicilin-like antimicrobial peptides 2-2-like	*Fragaria vesca subsp. Vesca*	1	1%	49	
324	Control	Protein folding	gi|166770	Heat shock protein 83	*Arabidopsis thaliana*	1	2%	66	
325	Control	Protein folding	gi|20559	hsp70 (AA 6–651)	*Petunia x hybrid*	2	5%	68	
326	Control	Protein folding	gi|20559	hsp70 (AA 6–651)	*Petunia x hybrid*	2	3%	54	
329	Control	Stress response	gi|226973436	Beta-thioglucoside glucohydrolase	*Carica papaya*	3	9%	165	
333	Control	Oxidation-redution process	gi|295367043	Cinnamoyl alcohol dehydrogenase	*Bambusa multiplex*	5	23%	48	
335	Control	Carbohydrate and enregy metabolism	gi|359483362	PREDICTED: lactoylglutathione lyase	*Vitis vinifera*	3	11%	146	
337	Control	Protein folding	gi|62433284	BiP	*Glycine Max*	2	5%	75	
339	Control	Stress response	gi|226973436	Beta-thioglucoside glucohydrolase	*Prunus pérsica*	3	6%	126	
340	Control	Stress response	gi|226973436	Beta-thioglucoside glucohydrolase	*Carica papaya*	3	6%	108	
343	Control	Fatty acid metabolism	gi|257096376	Full = GDSL esterase/lípase	*Carica papaya*	2	6%	163	
345	Control	Carbohydrate and energy metabolism	gi|356562473	PREDICTED: xylose isomerase-like	*Glycine Max*	3	9%	56	
347	Control	Other functions	gi|357440579	Pentatricopeptide repeat-containing protein	*Medicago truncatula*	7	12%	57	
**Proteins expressed similarly in both treatments**									
015	Control/PEG6	Stress response, metabolic process	gi|211906436	UDP-D-glucose pyrophosphorylase	*Gossypium hirsutum*	5	15%	213	
024	Control/PEG6	Other functions	gi|148912162	Cytosolic ascorbate peroxidase 1	*Gossypium hirsutum*	3	20%	221	
027	Control/PEG6	Oxidation-redution process	gi|449464176	PREDICTED: proteasome subunit beta type-3-A-like	*Cucumis sativus*	2	15%	175	
028	Control/PEG6	Metabolic process	gi|5669924	Soluble inorganic pyrophosphatase	*Populus tremula x Populus tremuloides*	1	7%	37	
032	Control/PEG6	Stress response	gi|226973430	Beta-thioglucoside glucohydrolase	*Carica papaya*	5	13%	278	
044	Control/PEG6	Unclassified	gi|412993224	ORF73	*Bathycoccus prasinos*	1	0%	36	
045	Control/PEG6	Other functions	gi|270313547	S-adenosylmethionine decarboxylase	*Olea europaea*	1	2%	47	
051	Control/PEG6	Protein metabolism, Stress response	gi|233955399	Calreticulin	*Carica papaya*	6	22%	323	
052	Control/PEG6	Oxidation-redution process	gi|193290660	Putative ketol-acid reductoisomerase	*Capsicum annuum*	3	10%	116	
056	Control/PEG6	Unclassified	gi|384249809	HI0933-like protein	*Coccomyxa subellipsoidea*	1	2%	58	
060	Control/PEG6	Oxidation-redution process	gi|449520553	PREDICTED: superoxide dismutase [Mn], mitochondrial-like isoform 1	*Cucumis sativus*	2	13%	204	
061	Control/PEG6	Oxidation-redution process	gi|33521626	Mn-superoxide dismutase	*Lotus japonicus*	1	8%	65	
062	Control/PEG6	Unclassified	gi|384253982	Hypothetical protein COCSUDRAFT_39121	*Coccomyxa subellipsoidea C-169*	1	1%	44	
067	Control/PEG6	Unclassified	gi|460412635	PREDICTED: 20 kDa chaperonin, chloroplastic-like	*Solanum lycopersicum*	3	12%	221	
069	Control/PEG6	Stress response, signaling	gi|2853219	Glutathione transferase	*Carica papaya*	2	10%	41	
087	Control/PEG6	Others functions	gi|55375985	14-3-3 family protein	*Malus x domestica*	6	33%	504	
095	Control/PEG6	Stress response	gi|357514981	Annexin-like protein	*Medicago truncatula*	2	5%	57	
099	Control/PEG6	Strress response	gi|356531939	PREDICTED: putative lactoylglutathione lyase-like	*Glycine Max*	3	9%	152	
126	Control/PEG6	Metabolic process	gi|5725356	Alpha-D-xylosidase	*Tropaeolum majus*	1	1%	37	
003	Control/PEG6	Unclassified	gi|357498189	DNA repair and recombination protein PIF1	*Medicago truncatula*	1	2%	40	
007	Control/PEG6	Oxidation-redution process	gi|12322163	Dormancy related protein, putative	*Arabidopsis thaliana*	1	3%	47	

PEG: polyethylene glycol.

^a^Spot numbers correspond to the numbers indicated in Figure 3.

^b^Maturation treatments: Control - without PEG; and PEG6 - with 6% PEG.

^c^Functional protein classification using Blast2go (http://www.blast2go.com).

^d^Accession number in the NCBI protein database.

^e^Number of unique peptide sequences identified by MASCOT.

^f^Percentage of predicted protein sequence covered by MASCOT.

^g^Probability-based MOWSE score from MASCOT software for the hit.

^h^Protein relative abundance according to the individual spot (%) volume determined using Image Master Platinum v.7 software (GE Healthcare). Change in abundance levels by t-test, *(p ≤ 0.05), **(p ≤ 0.01), ns (not significant).

**Figure 4:**
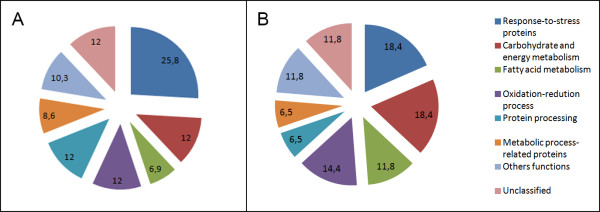
**Functional distribution (by Blast2GO) of all 76 proteins identified in the embryogenic cultures of *C. papaya *after 42 days of incubation in the (A) control without PEG and (B) PEG6 (with 6% PEG) maturation treatments.** PEG: polyethylene glycol.

The identified proteins (Table 2) were assigned to 7 different categories or were unclassified. This classification was performed by comparing the total numbers of proteins in each treatment, including those that were common to the two treatments (Table 2; Figure 4). The most representative class of proteins observed in the control treatment was associated with the stress response (25.8%), while those in the PEG6 treatment were related to carbohydrate and energy metabolism (18.4%) and the stress response (18.4%).

A comparative proteomic analysis between the control and PEG6 treatments was performed in papaya var. UENF/CALIMAN01, and the results are discussed based on the functional classifications of the differentially expressed proteins (Figure 4, Table 2).

### Response-to-stress proteins

We identified several stress-related proteins in the embryogenic cultures in both treatments (Figure 4, Table 2). Stress can force cells to switch to an embryogenic state, and recent proteomic studies have strongly emphasized the role of stress proteins during somatic embryogenesis [45]. The similarities of the proteins associated with the stress response identified in the two treatments in the present work indicate that embryogenesis, itself, is a stress inducer that is capable of stimulating various morphogenetic responses, as suggested by Pasternak et al. [46]. These stresses take effect during the regulation of the cell division cycle and reorganize the physiological, metabolic and gene expression patterns that occur during somatic embryo differentiation and development [47].

Glutathione transferase (spots 43 and 69), which was identified in both treatments at similar expression levels, is associated with stress tolerance and is induced by a wide array of biotic and abiotic stresses, including exposure to toxic chemicals, environmental stress and disease [48]. This enzyme was found to be up-regulated in cultures of *C. persicum* subjected to drought stress caused by transfer from a submerged suspension culture to a semisolid medium [49]. According to our results, the abundance of this protein was similar in the two treatments, indicating that other stresses in addition to water stress can regulate the activity and turnover of this protein during somatic embryo maturation in papaya var. UENF/CALIMAN01.

UDP-glucose pyrophosphorylase (spot 315) was identified in the control treatment, while UDP-D-glucose pyrophosphorylase (spot 15) was found at similar concentrations in the two treatments, and UDP-D-glucose pyrophosphorylase (spot 114) was found at the highest concentration in the PEG6 treatment. These proteins are essential enzymes for the synthesis of sucrose and other nucleotide sugars [50] that are precursors required for cell wall biogenesis [51]. In addition, many proteins involved in cell wall formation during normal development, including actin, are also recruited under stress conditions such as defense-related cell wall-remodeling events [52].

Methionine synthase (spot 128) and S-adenosylhomocysteine hydrolase (spot 237) were unique to the PEG6 treatment. Methionine synthase catalyzes the last step in the plant methionine biosynthesis pathway [53]. It provides methionine for protein synthesis in seeds and is stored throughout the entire process of seed maturation [54]. This protein has also been found in the mature dry seeds of *Arabidopsis*[55].

Other stress-related proteins identified in the papaya embryogenic cultures have few known functions in plants. For example, 2,3-bisphosphoglycerate-independent phosphoglycerate mutase (spots 17 and 139 ) is involved in plant defense responses, although its function has not yet been fully elucidated [56].

### Carbohydrate and energy metabolism proteins

Proteins associated with metabolism and the supply of energy were mainly identified in the PEG6 treatment, rather than the control treatment. These proteins play important roles during plant and embryo development as energy providers, acting in cofactor regeneration and the construction, interconversion and synthesis of metabolites, in addition to playing a role in signaling related to the regulation of various processes [57,58].

Enolase was the most abundant protein identified and was observed in five spots. Three of these spots (227, 243 and 244) were unique to the PEG6 treatment, and two spots (21 and 42) were common to the two treatments, with spot 21 being up-regulated by the PEG treatment (Table 2). Enolase is a ubiquitous enzyme that catalyzes the conversion of 2-phosphoglycerate to phosphoenolpyruvate, which is the only dehydration step in the glycolytic pathway, occurring at the end of glycolysis [59]. Enolase was the most frequently detected protein in the seeds of *C. persicum*, suggesting an important role of this protein in the embryogenesis of this species [60]. Similarly, enolase is the most abundant protein during late somatic embryogenesis in *Picea glauca*, suggesting that this protein can be used as a marker of embryo maturation [61]. During somatic embryogenesis in *Arabidopsis*, enolase transcript levels reach a maximum during the torpedo stage [62]. Another spot representing a glycolytic enzyme, corresponding to glyceraldehyde 3-phosphate dehydrogenase (spot 138) was found only in the PEG6 treatment.

Lactoylglutathione lyase proteins were identified in spot 335 in the control treatment and in spot 99 in both treatments. These enzymes are generally associated with cell proliferation [63] and might also be involved in antioxidation processes in tomato species [64].

### Proteins involved in fatty acid metabolism

Several proteins identified in the present work play a role in the fatty acid/lipid pathway. Several spots corresponding to GDSL esterase/lipase proteins (spots 11, 12 and 111) were identified in both treatments, being up-regulated in the control treatment, while one spot (343) was unique to the control treatment. Triosephosphate isomerase (spot 169), hypothetical protein VITISV_041523 (spot 175) and enoyl-ACP reductase (spot 194) were unique to the PEG6 treatment.

Papaya seeds are rich in lipids that are used during the germination process [34,65]. The greater number of proteins related to the fatty acid/lipid pathway that was observed in the PEG6 treatment (11.8%) compared with the control treatment (6.9%) (Figure 4) might indicate that water stress, which is inherent to this treatment, could be supporting the synthesis and mobilization of storage lipids during somatic embryogenesis.

### Proteins involved in oxidation-reduction processes

Most of the detected oxidation-reduction-related proteins were identified in both treatments, at similar expression levels. One exception was alcohol dehydrogenase 3 (ADH3) (spot 146), which was unique to the PEG6 treatment. ADH3 is involved in regulation of the effects of nitric oxide in the cell and is therefore important for the maintenance of cellular homeostasis [66]. Additionally, ADH3 has been reported to represent a class of enzyme that is closely related to the developmental stages of *Vitis rupestris* somatic embryos [67]. The other exception was cinnamoyl alcohol dehydrogenase (spot 333), which was unique to the control treatment. This enzyme catalyzes cinnamoyl CoA reductase, a key enzyme in the synthesis of lignin, which acts as a mechanical barrier to various plant pathogens [68].

The cytosolic ascorbate peroxidase 1 protein (spot 24) was observed in both treatments. Ascorbate peroxidases play a role in defense against oxidative stress [69]. This protein functions in the defense against photooxidative stress in *Arabidopsis*[70]. In the present work, the presence of this protein in both treatments provided evidence of a response to the stress caused by the *in vitro* culture conditions.

Reactive oxygen species (ROS) play an important role during plant growth and development, as they are extremely reactive in regulating hormonal activity [71]. The activity of ROS is often the first detectable response to biotic or abiotic stress in plants [72]. A large number of studies have shown increases in the levels of ROS during somatic embryo development [73]. ROS play a role as secondary messengers involved in signal transduction, thereby influencing the expression of many genes [74].

This similarity between the two treatments related to the identified proteins and their relative expression levels could be associated with the oxidative stress inherent to the *in vitro* culture conditions.

### Protein processing

Many heat shock proteins (HSPs) were identified during papaya embryogenesis. The HSP70 (spots 325 and 326) and HSP83 (spot 324) proteins were unique to the control treatment, while HSP90 (spot 162) was unique to the PEG6 treatment. The HSP70 protein (spot 49) was observed in both treatments (Table 2).

The HSP70 family consists of chaperone proteins that bind to exposed hydrophobic sequences and maintain the unfolded peptide chain until it folds into the correct three-dimensional conformation [75]. HSP70 proteins are responsible for the translocation and processing of proteins associated with reserve synthesis and mobilization [76], abiotic stress [77] and cellular defense [78]. In addition, HSP70s have been associated with seed development in *Dimocarpus longana*[79] and somatic embryo development in *Vitis vinifera*[72] and *Picea abies*[80].

HSP83 was unique to the control treatment. The function of this protein family is still unknown. However, HSP83 has been detected during the stress response to *Ustilago maydis* infection in *Z. mays*[81].

The members of the HSP90 family, which was unique to the PEG6 treatment, are involved in the assembly, maturation, stabilization and activation of proteins that are important as signaling molecules, such as protein kinases, hormone receptors and transcription factors, in eukaryotic cells [82]. They are responsible for refolding denatured proteins and/or folding newly synthesized proteins [83]. These proteins have also been identified during somatic embryogenesis in hybrid larch (*Larix* × *eurolepis*) [84] and in the developing seeds of *Pinus massoniana*[85] and *Araucaria angustifolia*[86].

BPI (spot 337), which was unique to the control treatment, is a member of the HSP70 family and localizes to the endoplasmic reticulum. It acts in the translocation of proteins through the endoplasmic reticulum and assists in the proper folding and maturation of newly synthesized proteins entering the organelle. Therefore, this protein has been implicated in seed maturation in several species, such as *Cucurbita maxima*[87], *Ocotea catharinensis*[88] and *Pinus massoniana*[85]. However, overexpression of this protein inhibits the accumulation of seed storage proteins in *Oryza sativa* endosperm cells [89].

A leucine aminopeptidase (spot 142) was found to be unique to the PEG6 treatment. During plant development, proteases exhibit important functions, including the degradation of damaged, misfolded and potentially harmful proteins to provide free amino acids required for the synthesis of new proteins [90]. The observation of this protein only in the PEG6 treatment could be indicative of better control of embryonic development under this treatment.

### Metabolic process-related proteins

A high percentage of metabolic process-related proteins was observed in both treatments, and the expression levels of these proteins were similar in most cases (Table 2).

Among these proteins, pyrophosphatase (spot 28), which was common to the two treatments, is a metalloenzyme that catalyzes diverse reactions that are necessary to supply the energy required during various biosynthetic reactions [91]. This protein is associated with stress tolerance [92]. Another protein that was common to both treatments, alpha-D-xylosidase (spot 126), mobilizes xyloglucan during the development of germinated cotyledons in nasturtium (*Tropaeolum majus*) seeds [93]. Aldehyde dehydrogenase (spot 130) was unique to the PEG6 treatment. Despite being involved in various functions, there is evidence that this protein may play a role in seed desiccation tolerance and vigor [94], as reported in rice [56]. The possible relationship of this protein to desiccation tolerance may explain its expression only in the PEG6 treatment.

The PEG6 treatment also induced the synthesis of the unique protein esterase D (spot 184). This protein is considered a biomarker of competent embryogenic cultures in *D. carota*[95] and *Z. mays*[96]. Esterase D is also involved in various cellular functions, such as fruit ripening, abscission, cell expansion, stomatal movement, reproduction and the detoxification of xenobiotics [97].

### Proteins related to other functions

Some functional categories contained only a few proteins that were identified in the papaya embryogenic cultures (Table 2). Among these proteins, the initiation factor eIF4A-15 was observed as a unique spot in both treatments (spot 222 and spot 317 in the PEG6 and control treatments, respectively). This protein phosphorylates other proteins involved in translation, allowing protein translation to be coupled with other essential cellular functions, such as the stress response, cell growth and division. Proteins related to changes in the state of chromatin transcription in genes via DNA processing, mRNA translation and post-translational modifications are responsible for regulating the levels of protein expression for several genes [98]. This protein is likely involved in the common, inherent process of somatic embryogenesis in papaya, as it is not dependent on the induction of maturation treatments.

A 14-3-3 protein (spots 48 and 87) that was common to both treatments is an important type of cellular signaling protein that regulates enzymes during primary metabolism and performs other ‘housekeeping’ functions [99]. In addition, this protein participates in the regulation of various biochemical processes during seed development [100].

S-adenosylmethionine decarboxylase (spot 45) was observed in both treatments (Table 2). This protein is a key enzyme involved in the synthesis of polyamines in plants and other organisms [101]. Polyamines play a role in several processes in plants and have been related to the development of somatic embryogenesis [102].

## Conclusions

Since the introduction of somatic embryogenesis [103,104], a series of experiments have been performed in an attempt to elucidate the molecular, physiological and biochemical regulation of the morphogenic competence pathways in plant cells. Nevertheless, one key scientific question concerning somatic embryogenic development remains: “How does a single somatic cell become a whole plant?” [105]. The field of proteomics could be at the heart of such research questions, and it has become increasingly important in studies on somatic embryogenic development.

This is the first report describing a proteomic analysis of somatic embryogenesis in papaya. Our results showed that it was possible to detect a pattern of differential protein expression during somatic embryo differentiation and maturation in papaya induced by PEG6 treatment. PEG (6%) treatment promoted increased protein synthesis and induced differential expression of proteins related to carbohydrate and energy metabolism, fatty acid metabolism and oxidation-reduction in embryogenic cultures compared to the control treatment.

We observed that the synthesis of certain proteins induced by PEG treatment, such as enolase, esterase and ADH3, could play an important role in the maturation of somatic papaya embryos. These proteins have been found to be associated with somatic embryo development in other species [60,61,67,106], and esterases are considered important biomarkers of somatic embryos in plants [106]. Additionally, the present study provides a list of PEG-induced proteins that are potential biomarker candidates for future investigations of somatic embryogenic development in papaya.

## Materials and methods

### Plant material

To induce somatic embryogenesis in papaya, immature zygotic embryos were isolated from mature papaya seeds of the hybrid UENF/CALIMAN01 and used as explants. Immature fruits were kindly provided by the Caliman Agricola S/A company, which is located in the city of Linhares, Espírito Santo (ES), Brazil (19° 23’S and 40° 4’W).

### Induction and multiplication of embryogenic cultures

The induction of embryogenic cultures was performed according to Heringer et al. [20]. Briefly, immature fruits were disinfected for 2 min in 70% ethanol and for 30 min in 50% commercial bleach, followed by three washes with distilled, autoclaved water. Immature seeds were subsequently obtained and sorted in a laminar flow cabinet, and immature embryos were isolated for use as explants. The immature embryos were inoculated into tubes (25 × 150 mm) containing 10 mL of MS culture medium [107] (Phytotechnology Lab, Shawnee Mission, KS, USA) supplemented with 3% sucrose (Vetec, São Paulo, Brazil), 20 μM 2,4-dichlorophenoxyacetic acid (2,4-D) (Sigma-Aldrich, St. Louis, USA) and 2.0 g/L Phytagel (Sigma-Aldrich). The pH of the culture medium was adjusted to 5.8 before the Phytagel was added. The culture medium was sterilized via autoclaving at 121°C for 15 min. After inoculation, the tubes with the explants were incubated in the dark at 25°C (±2°C) for 42 days. The induced embryogenic cultures were then isolated and subcultured in a culture medium with the same composition. Four subcultures were then obtained at intervals of 21 days prior to maturation to perform multiplication of embryogenic cultures.

### Maturation of embryogenic cultures

After the multiplication phase, the cultures were transferred to maturation treatments. Three colonies, each with a fresh mass (FM) of 300 mg, were inoculated into Petri dishes (90 × 15 mm) containing 20 mL of MS culture medium supplemented with myo-inositol (Merck KGaA, Darmstadt, Germany) (0.005%), Phytagel (2.0 g/L), sucrose (3%) and PEG 3350 (Sigma-Aldrich) at concentrations of 0, 3, 6 and 9%, hereafter referred to as the control, PEG3, PEG6 and PEG9 treatments, respectively. The pH of the culture medium was adjusted to 5.8 before the Phytagel was added. The culture medium was sterilized by autoclaving at 121°C for 15 min. The cultures were incubated in a growth chamber at 25 ± 1°C in the dark for the first 7 days, after which they were subjected to a 16 h light (60 μmol/m^2^ s^1^) photoperiod. Four repetitions were performed, with a total of 12 colonies per treatment. After 42 days of cultivation, the number of cotyledonary somatic embryos (mature somatic embryos) per colony was evaluated. Samples of 300 mg (FM) were dried in an oven at 70°C for 48 h for dry matter (DM) determination. Samples of 300 mg FM were also stored at -20°C for proteomic analysis.

### Preparation for microscopy

The samples were fixed in an aqueous solution containing glutaraldehyde (2.5%) and formaldehyde (4.0%) diluted in sodium cacodylate buffer (0.1 M), pH 7.3, at room temperature for 24 h. After fixation, the samples were dehydrated in a graded ethanol series and embedded in historesin (Leica, Wetzlar, Germany) [108]. Sections with a thickness of 3 μm were cut with a microtome (Leica) and fixed on slides by heating.

The samples were dehydrated with periodic acid and stained via the periodic acid-Schiff reaction. Protein bodies were stained with Coomassie brilliant blue R-250 (CBB) [109]. The samples were observed under an Axioplan light microscope (Carl Zeiss, Jena, Germany) equipped with an Axiocam MRC5 digital camera (Carl Zeiss), and the images were analyzed using AxioVisionLE version 4.8 software (Carl Zeiss).

### Protein extraction

Protein extracts were prepared in biological triplicate (300 mg FM) for each maturation treatment. Soluble proteins were extracted according to the method described by Santa-Catarina et al. [110]. The buffer-soluble proteins were extracted with phosphate buffer (pH 7.5) containing 50 mM sodium phosphate dibasic (Vetec), 10 mM 2-mercaptoethanol (Sigma-Aldrich) and 1 mM phenylmethylsulfonyl fluoride (PMSF) (Sigma-Aldrich). The supernatants were transferred to clear microtubes, and the proteins were precipitated on ice for 30 min with 10% trichloroacetic acid (Merck). The pellet was washed three times with cold acetone (Merck), and the proteins were then resuspended and concentrated in 0.5 mL of a urea/thiourea buffer (7 M urea, 2 M thiourea, 1% dithiothreitol (DTT), 2% Triton X-100, 0.5% pharmalyte (all from GE Healthcare, Freiburg, Germany) and 1 mM PMSF), to which a 0.5% immobilized pH gradient (IPG) buffer (pH 4–7) (GE Healthcare) was added. The protein concentration was determined using the 2-D Quant Kit (GE Healthcare).

### Two-dimensional gel electrophoresis (2-DE) and spot matching

The 2-DE analyses were performed in the protein extracts from the embryogenic cultures from both the control (the treatment that resulted in the fewest mature embryos) and PEG6 (the treatment resulting in the greatest number of somatic embryos) treatments to compare these two conditions. Embryogenic cultures were used, once the isolation of somatic embryos in different developmental stages was unfeasible, due not have sufficient material for the extraction of the amount of protein required for the initial stages of development.

Aliquots of the samples containing 500 μg of protein were used for 2-DE. Prior to loading onto 18 cm IPG strips (pH 4 – 7), a sufficient volume of rehydration buffer (7 M urea, 2 M thiourea, 2% 3-[(3-cholamidopropyl) dimethylammonio]-1-propanesulfonate (CHAPS) (GE Healthcare), 0.5% IPG buffer, pH 4–7, 1% DTT and 0.002% bromophenol blue (Sigma-Aldrich)) was added to the sample aliquots to achieve a final volume of 375 μL. After 12 h in the rehydration gel, isoelectric focusing was performed using an IPGphor II apparatus (GE Healthcare) for a total of 35 kVh at 20°C. The IPG strips were then subjected to reduction and alkylation through 2 × 15 min incubations, using a buffer (50 mM Tris–HCl (GE Healthcare), 6 M urea, 30% glycerol (Sigma-Aldrich), 2% sodium dodecyl sulfate (SDS) (Sigma-Aldrich) and 0.002% bromophenol blue) containing 125 mM DTT for the first incubation and 125 mM iodoacetamide (GE Healthcare) for the second incubation. Then, the strips were applied to the top of a 12% polyacrylamide gel. The second dimension of electrophoresis was performed at 25 mA per gel using a Protean II apparatus (Bio-Rad, Hercules, USA), and the gels were stained with Coomassie (0.1% Coomassie brilliant blue G250, 1.2% ortho-phosphoric acid (85%) and 10% ammonium sulfate) according to Neuhoff et al. [111].

The Coomassie-stained 2-DE gels obtained for each of the three biological samples were analyzed using Image Master Platinum v.7 software (GE Healthcare). The authenticity and the outline of each protein spot was validated via visual inspection and edited when necessary. The identification and selection of the differentially expressed proteins were achieved through comparative analysis of the gels, and the volumes of individual spots were obtained following the program’s instructions. To eliminate gel-to-gel variations, the individual spot volume in each gel was normalized relative to the total valid spot volume, expressing the protein abundance as the relative volume (%vol), and the values obtained for the control and PEG6 treatments were compared using Student’s t-test. The 135 most abundant proteins present in three biological replicates, 21 of which were unique to the control, while 47 were unique to PEG6, and 67 were common to both treatments, were selected for MS/MS identification.

### In-gel digestion and tandem mass spectrometry (MS/MS) analysis

Protein identifications were obtained through in-gel digestion and matrix-assisted laser desorption/ionization time-of-flight (MALDI-TOF/TOF)-MS/MS. The protein spots were manually excised from the 2-DE gels and washed with water. The spots were then selected according to the methodology proposed by Balbuena et al. [86] and digested with trypsin (Promega, Madison, WI, USA). The resulting peptides were concentrated and desalted using C18 Zip Tips (Millipore). The final solutions of extracted peptides were stored at -20°C until MS/MS analysis.

Mass spectrometry analysis was performed according to Rocha et al. [112] and Gandra et al. [113]. Briefly, 0.3 μL of the sample solution was mixed with an equal volume of a saturated matrix solution [10 mg/mL α-cyano-4-hydroxycinnamic acid (Sigma-Aldrich) in 50% acetonitrile (Merck)/0.1% trifluoroacetic acid (Sigma-Aldrich)] on a target plate and allowed to dry at room temperature. Raw data for protein identification were obtained using an AB SCIEX TOF-TOF 5800 mass spectrometer (Applied Biosystems, North Warrington, Cheshire, United Kingdom). External calibration was performed in MS mode using a mixture of four peptides: des-Arg1-bradykinin (*m/z* 904.468); angiotensin I (*m/z* 1296.685); Glu1-fibrinopeptide B (*m/z* 1570.677); and ACTH (18–39) (*m/z* 2465.199) (all from Applied Biosystems). The MS/MS spectra were externally calibrated using the known product ion masses observed in the MS/MS spectrum of angiotensin I.

### Database searches

All MS/MS samples were analyzed using Mascot (Matrix Science, http://www.matrixscience.com). Mascot was set up to search the NCBInr database (selected for Viridiplantae) assuming the use of the digestion enzyme trypsin, with up to two missed cleavages. Mascot was searched with a product ion mass tolerance of 0.30 Da and a precursor ion tolerance of 0.60 Da. Methionine oxidation and the iodoacetamide derivative of cysteine were specified as variable and fixed modifications, respectively. Only significant hits, as defined through MASCOT probability analysis (P < 0.05), and peptides identified with individual ion scores greater than 30 were considered.

Functional classification of the proteins identified by Mascot was performed using the program Blast2go (http://www.blast2go.com).

## Abbreviations

2,4-D: 2,4-dichlorophenoxyacetic acid; 2-DE: Two-dimensional gel electrophoresis; ABA: Abscisic acid; ADH3: Alcohol dehydrogenase 3; CHAPS: 3-[(3-cholamidopropyl) dimethylammonio]-1- propanesulfonate; DTT: Dithiothreitol; FM: Fresh mass; IPG: Immobilized pH gradient; HSP: Heat shock proteins; MALDI-TOF/TOF: Matrix-assisted laser desorption/ionization time-of-flight; MS: Murashige and Skoog; MS/MS: Tandem mass spectrometry; NCBI: National Center for Biotechnology Information; PEG: Polyethylene glycol; PEG3: Polyethylene glycol 3%; PEG6: Polyethylene glycol 6%; PEG9: Polyethylene glycol 9%; PMSF: Phenylmethylsulfonyl fluoride; SDS: Sodium dodecyl sulfate.

## Competing interests

There are no competing interests in this study.

## Authors’ contributions

EMV, CSC and VS conceived the study and designed the experiments. EMV, VS, ASH and CSC wrote the manuscript. ASH conducted two-dimensional gel electrophoresis and spot matching. ATSF, MNC, TB and JEAP were responsible for the in-gel digestion and tandem mass spectrometry (MS/MS) analysis and database management. All authors read and approved the final manuscript.
